# The Impact of Travel Time on Geographic Distribution of Dialysis Patients

**DOI:** 10.1371/journal.pone.0047753

**Published:** 2012-10-17

**Authors:** Saori Kashima, Masatoshi Matsumoto, Takahiko Ogawa, Akira Eboshida, Keisuke Takeuchi

**Affiliations:** 1 Department of Public Health and Health Policy, Hiroshima University Institute of Biomedical and Health Sciences, Hiroshima, Japan; 2 Department of Community-Based Medical System, Faculty of Medicine, Hiroshima University, Hiroshima, Japan; 3 Centre for Kidney Diseases, Hiroshima Prefectural Hospital, Hiroshima, Japan; Vanderbilt University, United States of America

## Abstract

**Backgrounds:**

The geographic disparity of prevalence rates among dialysis patients is unclear. We evaluate the association between travel time to dialysis facilities and prevalence rates of dialysis patients living in 1,867 census areas of Hiroshima, Japan. Furthermore, we study the effects of geographic features (mainland or island) on the prevalence rates and assess if these effects modify the association between travel time and prevalence.

**Methods:**

The study subjects were all 7,374 people that were certified as the “renal disabled” by local governments in 2011. The travel time from each patient to the nearest available dialysis facility was calculated by incorporating both travel time and the capacity of all 98 facilities. The effect of travel time on the age- and sex-adjusted standard prevalence rate (SPR) and 95% confidence intervals (CIs) at each census area was evaluated in two-level Poisson regression models with 1,867 census areas (level 1) nested within 35 towns or cities (level 2). The results were adjusted for area-based parameters of socioeconomic status, urbanity, and land type. Furthermore, the SPR of dialysis patients was calculated in each specific subgroup of population for travel time, land type, and combination of land type and travel time.

**Results:**

In the regression analysis, SPR decreased by 5.2% (95% CI: −7.9–−2.3) per 10-min increase in travel time even after adjusting for potential confounders. The effect of travel time on prevalence was different in the mainland and island groups. There was no travel time-dependent SPR disparity on the islands. The SPR among remote residents (>30 min from facilities) in the mainland was lower (0.77, 95% CI: 0.71–0.85) than that of closer residents (≤30 min; 0.95, 95% CI: 0.92–0.97).

**Conclusions:**

The prevalence of dialysis patients was lower among remote residents. Geographic difficulties for commuting seem to decrease the prevalence rate.

## Introduction

Although dialysis is increasingly available among the population and the rate of persons undergoing dialysis has been increasing around the world [1], there is striking inequity in usage rate of dialysis among socioeconomic groups [2]–[5]. Geographic inequity in prevalence of dialysis, however, is less well known because there are few pertinent epidemiological studies on the topic, particularly in non-European countries [4]–[8].

Geographic inequity of dialysis care certainly exists. Japan has a high rate of patients undergoing dialysis: 227.9 per 100,000 people in 2009 [9], [10]. This value is much higher than that of the United States (125.7) or United Kingdom (UK) (41.7) [1]. In addition to the very low rate of renal transplantation among end-stage renal disease patients in Japan [11], universal health insurance coverage [12] and special financial support for dialysis patients from public expenses are likely major contributors to this high prevalence. The copayment for maintenance haemodialysis therapy is usually completely exempted or reduced by half of the copayment price depending on the household income. However, despite this economically egalitarian health system for dialysis patients, there is geographic inequity in dialysis care provision and this problem has received no political attention. There is no public policy to intervene with health resource distribution such as facilities and human resources. Consequently, a majority of facilities and medical staff–particularly physicians–are concentrated in urban areas [13]. Density of nephrologists per 100,000 people was substantially different across prefectures of Japan as there are 5.3-times differences between the lowest and highest prefectures [14].

In addition, racially, Japan is close to being a homogeneous country [15]. The racial homogeneity and egalitarian health economic system in Japan provides a great opportunity to observe the effect of geography on dialysis service utilization, as race and the financial burden of healthcare are essentially equal for all subjects. The effect would be less biased by racial and economic variability among the population in Japan compared with countries such as the United States and UK [2]–[5].

In this study, we evaluate the geographic variability in prevalence rates of dialysis patients among census areas in Hiroshima prefecture, Japan, with particular focus on the association between the prevalence and the travel time to available facilities.

## Methods

### Study Areas

**Figure 1:**
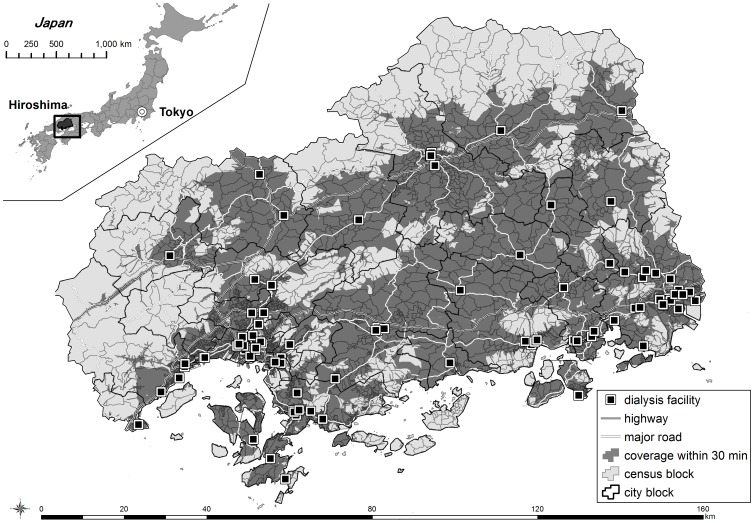
Map of Hiroshima prefecture with road network, coverage within 30 min from dialysis facility.

Hiroshima prefecture is located in the western part of Japan (Figure 1). Its population was 2,860,750 according to the 2011 vital census. The number of nephrologists in Hiroshima was 4.6 per 100,000 people.

For the area-based analysis, we employed the second-smallest census block, which is smaller than a municipality (city, town or village). There are 1,869 census blocks in Hiroshima, and we excluded 2 blocks because of lack of population data by age group.

### Dialysis Patients

The study subjects were 7,374 first- and third-grade “renal disabled” patients certified by municipal governments, which includes all age groups. The postal codes of all the certified persons were obtained on 1 August 2011 from all the 23 municipality governments (capture rate 100%). The first- and third-grade renal disabled patients are required, by definition, to have their serum creatinine levels no less than 5.0 mg/dL, or creatinine clearance less than 20 mL/min [16].

The certified renal disabled are entitled to receive extra financial support for dialysis therapy from public expenses. Copayment by the certified patients for their dialysis therapy, which is 10,000 to 20,000 Japanese yen (equivalent to 125 and 295 US dollars), per month without these certifications, are reduced to total exemption or half of the normal price according to their household’s income. Moreover, the renal disabled can receive other non-medical benefits such as reduced public transportation fees for commuting to dialysis facilities. Due to this generous financial support, most patients with renal disease in Japan apply for certification of first- or third-grade renal disability when they begin dialysis therapy. As a preliminary survey, we checked the certified disability status among all the dialysis patients as of June 2011 at seven medical institutions (three in the capital city of Hiroshima and four in northern rural areas). Of the 486 dialysis patients at the institutions, 483 (99.3%) were certified as first- or third-grade renal disability.

### Dialysis Facilities

There are 98 dialysis facilities in the study area. The address, number of dialysis units, and the maximum number of outpatients (capacity) of 90 facilities were obtained from the Japanese Society for Dialysis Therapy. The information for the other 8 facilities was directly obtained from each facility. The capacity was the number of commuting patients at each facility, which the director of the facility considered as the maximum number accounting for the available number of dialysis units and human resources.

### Measures for Accessibility

The details are described elsewhere [17], so the modeling approach adopted is shown briefly. Initially, the postal codes of study subjects were geocoded. We excluded 7 patients because their addresses were located in another prefecture. The addresses of 98 dialysis facilities located in the study were also geocoded. We then calculated the travel time by car from each patient to a dialysis facility in two models: 1) The closest facility is regarded as the facility the patient commutes to (distance model); and 2) identifies commuting facility taking into account both travel time and capacity of each facility (capacity-distance model).

The travel time in the capacity-distance model was calculated as follows: First, each facility accepted patients in order of shorter travel-time until it reached the limit of its capacity. Second, if a patient was not accepted by a facility in the first step, the patient approached the next-nearest facility in the same manner as the first step. Then, it ran through the first and second steps until all the patients were accepted by any one of the facilities. In the calculation process, we carried out network analysis (i.e., found the shortest travel-path between two locations on a road network, including highways), by using geographic information system software ArcGIS version 10.0 and ArcGIS Data Collection Road Network 2011 (ESRI Japan Inc.). In the Road Network, driving speeds of all the segments of the roads are classified into 14 categories depending on the type and width of the segment. Time of ship travel, including travel time to ferry port in island and waiting time (10 min), was added to commuting times of patients in the island without any bridge connection to the mainland.

### Measurement of Area-based Characteristics

Data of population by sex and 5-year age group and of the proportion of tertiary industry workers among the residents ≥15-years-old (the age category officially used in Japan) at each census block were obtained from the 2005 National Census. According to the Japan standard industrial classification, the tertiary industry includes various service-related occupations such as transportation/communications, wholesale/retail trade, finance/insurance, and civil services. The full description of occupational groups is available on-line in English [18].

As an area-based parameter of urbanity/rurality, the population density was calculated in each census block. As an area-based geographic characteristic, we divided the area into mainland and island. Some residents in islands need additional transportation measures (e.g., ship) to reach dialysis facilities. This can potentially affect accessibility and thus, the mainland-island categories were created.

### Statistical Analysis

First, we evaluated the effect of a 10-minute increase of travel time on age- and sex-adjusted standard prevalence ratio (SPR) at each census block using the multilevel log-normal Poisson regression model with a random intercept [19], [20]. This model allows intercept to vary across geographic localities since our data covers the whole prefecture with wide variations in terms of regional characteristics (e.g., different public transportation services or different climatic conditions). The data had a two-level structure of 1,865 census blocks at level 1 nested within 35 town or city blocks at level 2. An offset term is entered to allow for the size of age- and sex-group of population in each census block. Travel time and all covariables were treated as level 1 variables. First we examined the crude association between the travel time and observed number of dialysis patients (crude model); we then adjusted the association accounting for the rate of tertiary industry workers, population density, and type of land (adjusted model). The fixed and random parameter estimates were calibrated using a predictive quasi-likelihood procedure with second-order Taylor series expansion, as implemented using the multilevel modeling software MLwiN 2.24 (Centre for Multilevel Modeling, Bristol University 2011) [21]. For all analyses, a P-value <0.05 (two-sided) was considered statistically significant. To determine whether the effect of travel time is influenced by land type, a test of interaction was conducted by including interaction terms between the travel time and land type in both crude and adjusted models. If interaction term was significant, we stratified the participants into two groups in each land type. We then estimated the mean predicted age- and sex-adjusted SPR for dialysis patients by a two-level, multilevel model including interaction effect between travel time and land type. In the interaction model, as well as in the model without interaction, we adjusted the association for a rate of workers in tertiary industry and population density at each census block.

Finally, as specific SPR analysis, SPRs of dialysis patients and their 95% confidence intervals (CIs) in both time and capacity-distance models were calculated in each of the following specific subgroup of population: 1) Whole study area; 2) the land type (mainland or island); 3) the travel time (≤30 min and >30 min); and 4) combination of land type and travel time. We calculated the SPR using the prevalence of dialysis patients in Japan in 2010 as the reference [9], [22]. We estimated the 95% CI using Wald method for each SPR, assuming that the number of observed dialysis patients were under a Poisson distribution [23]. Furthermore, by using the estimated travel time-specific SPR (≤30 min and >30 min), which we calculated in specific SPR analysis, we calculated a population-attributable risk percent of prevalence for travel time. We then estimated the number of patients that would have emigrated from distant areas if the effect of travel time on SPR had been solely due to the emigration.

In addition, we created a map of age- and sex-adjusted SPRs of dialysis patients in all census blocks. In some census blocks, as a geographic unit, SPRs were too unstable to be calculated due to the small numbers of patients. Thus, the empirical Bayesian estimation [20] was adopted and the eBayes function [24] was used in R version 2.14.1 (R development Core Team 2011) [25].

As a sensitivity analysis, we adopted another capacity-distance model for calculating travel time in which patients were accepted by a facility according to the descending order of travel time in the second step (i.e., the facility preferably accepted remote patients in its catchment area). We then tested the robustness of the effect of travel time on SPR using the two capacity-distance models.

The data in this study was collected by local governments and used in anonymous form with permissions from the governments. This study was approved as a study that can be conducted without individual informed consent by the Ethics Committee of Epidemiological Research, Hiroshima University.

## Results

**Figure 2:**
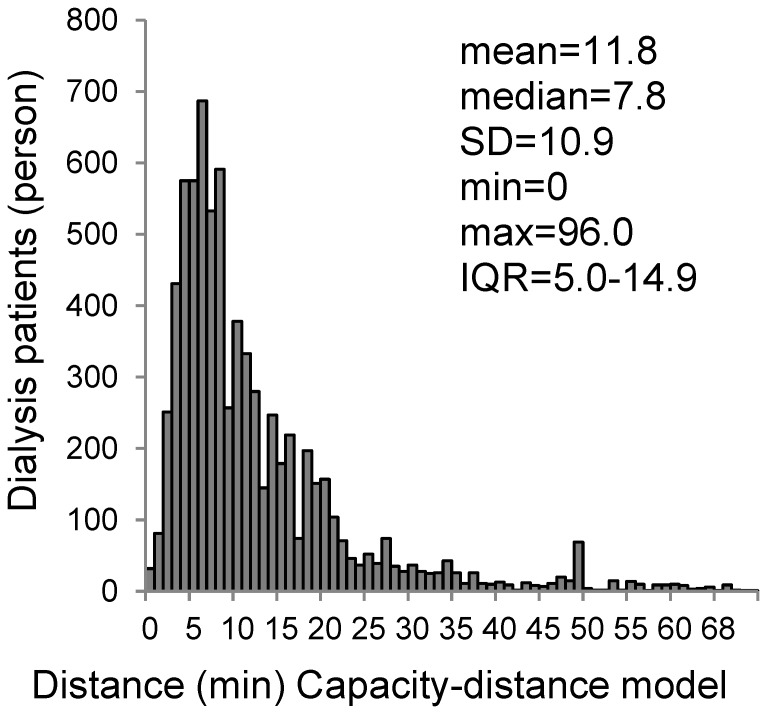
Distribution travel times of dialysis patients in capacity-distance model. IQR, interquartile range; SD, standard deviation.

Table 1 shows the demographic, social and environmental characteristics of census blocks sorted according to travel time to the closest (or closest available) dialysis facility. About 6% of the population lives in areas with travel times longer than 30 min. These remote areas had a higher rate of people age 65 or older and a lower rate of workers in tertiary industry than close areas (≤30 min). In addition, almost a quarter of them (23%) lived on islands. The distribution of dialysis patients in travel time in the capacity-distance model is shown in Figure 2. The median travel time in the capacity-distance model was 7.8 min (min: 0 and max: 96 min).

**Table 1:** Demographic, social and environmental characteristics of 1,867 census blocks classified according to travel time to the closest dialysis facility in 2011 in Hiroshima, Japan.

		Distance model		Capacity-distance model	
	unit [scale]	≤30 min	>30 min	≤30 min	>30 min
**Census block**	number [block]	1,765	102	1,614	253
**Population**	median [person]	834	192	892	374
	(IQR)	(330–1,944)	(89–479)	(341–2,034)	(147–820)
**Age group**					
Age 0 to 14	number (%)^a^ [person]	399,685 (14)	3,586 (9)	383,401 (14)	19,870 (12)
Age 15 to 64		1,839,587 (65)	19,262 (50)	1,758,308 (65)	100,541 (59)
Age over 65		585,235 (21)	15,310 (40)	550,129 (20)	50,416 (30)
**Population density**	median [per km^3^]	1,684	19	2,187	50
	(IQR)	(104–5,971)	(10–74)	(129–6,333)	(18–201)
**Rate of workers in tertiary industry**	median [%]	86	53	87	62
	(IQR)	(74–90)	(42–64)	(76–90)	(49–79)
**Land type**					
Mainland	number (%)^a^ [block]	1674	(95)	84	(81)
Island		89	(5)	20	(19)

IQR: interquartile range.

a Percentages were calculated by dividing the number of each group by total number in the corresponding travel time-category.

Table 2 shows the percentage change in age- and sex-adjusted SPR ratio for dialysis patients per 10 min increase in travel time. The SPR ratio in the adjusted model significantly decreased by 5.5% (95% CI: −8.3–−2.7) with each 10-minute increase in travel time. This means that there is a linear association between travel time and dialysis prevalence. Interaction term was significant between travel time and land type, which means there is a synergetic association between these two variables. Figure S1 shows the mean predicted age- and sex-adjusted SPR for dialysis patients per 10-min increase in travel time in the capacity-distance model, stratified by land type. Although the linear association between travel time and SPR is clearly observed in the mainland, the predicted SPR among residents in islands did not change with the increase in travel time.

**Table 2:** Percent change in age- and sex-adjusted standard prevalence rate (SPR)^a^ ratio for dialysis patients per 10-min increase in travel time to the nearest dialysis facility (census block *n* = 1,867).

	Distance model			Capacity-distance model		
	Percent change	95% CI	*P-*value for interaction between travel timeand land type^b^	Percent change	95% CI	*P-*value for interaction between travel time and land type^b^
Crude model	−13.2	(−16.6–−9.8)	<0.01	−7.4	(−9.7–−5.0)	<0.01
Adjusted model^c^	−10.8	(−14.9–−6.5)	<0.01	−5.2	(−7.9–−2.3)	<0.01

CI: 95% confidence interval.

a SPR means that prevalence rate of dialysis patients in each census block was adjusted by using the distribution of the age and sex of the census block.

b A significance of interaction (*P*<0.05) between travel time and land type means that there is a synergetic association between these two variables.

c Adjusted for rate of tertiary industry workers in census block, population density, and geographic type (mainland or island).

The age- and sex-adjusted SPR of dialysis patients in each travel time and land type group is shown in Table 3. The SPR of the whole study area was 0.93 (95% CI: 0.91–0.96). A lower SPR was observed for islands (0.86, 95% CI: 0.78–0.93) compared to the mainland (0.94, 95% CI: 0.92–0.96). Compared with the SPR among closer residents (≤30 min) in the capacity-distance model (0.95, 95% CI: 0.92–0.97), the SPR among remote residents (>30 min) was lower (0.77, 95% CI: 0.71–0.85). These point estimates indicate that dialysis is less common among remote residents compared with those living within 30 min. The non-overlapping confidence intervals strengthen this interpretation. This effect of travel time was not only observed in the whole prefecture, but also in the mainland. The SPR among remote residents was lower (0.72, 95% CI: 0.64–0.81) than the SPR among closer residents (0.95, 95% CI: 0.93–0.98) on the mainland. It is noted, however, that there is no travel time-dependent SPR difference among residents in islands. The SPR in remote islands was higher than that in remote areas of the mainland, while the SPR in non-remote islands was lower than that in their mainland counterpart.

**Table 3:** Number, age- and sex-adjusted standard prevalence rate (SPR) and their 95% CIs of dialysis patients in each subgroup of population in 2011 in Hiroshima, Japan (total population *n* = 2,828,506 and census block *n* = 1,867 for the analysis).

	ALL			Distance model (mean: 12.1, IQR: 5.4–15.9)			Capacity-distance model (mean: 15.5, IQR: 5.6–19.6)		
	Obs.	SPR	(95% CI)	Obs.^a^	SPR	(95% CI)	Obs.^a^	SPR	(95% CI)
**Entire population**	7,381	0.93	(0.91–0.96)						
**Land type**									
Mainland	6863	0.94	(0.92–0.96)						
Island	502	0.86	(0.78–0.93)						
**Travel time category**									
≤30 min				7,210	0.93	(0.91–0.96)	6,894	0.95	(0.92–0.97)
>30 min				155	0.85	(0.73–1.00)	471	0.77	(0.71–0.85)
**Land type-travel time category**									
Mainland									
≤30 min				6801	0.94	(0.92–0.97)	6573	0.95	(0.93–0.98)
>30 min				62	0.65	(0.50–0.83)	290	0.72	(0.64–0.81)
Island									
≤30 min				409	0.82	(0.74–0.90)	321	0.84	(0.76–0.94)
>30 min				93	1.08	(0.88–1.32)	181	0.88	(0.76–1.02)

IQR, interquartile range; Obs, observation number of dialysis patient; SPR, standard prevalence rate; CI: 95% confidence interval.

a Total observation number was smaller than the total observation number of all populations due to lack of population numbers in census block.

When the difference in SPR is considered to be caused solely by patient emigration, the estimated number of patients that moved from distant to closer areas was calculated as 83 based on the gap between the expected prevalence (558 patients), which is calculated assuming the effect of travel time on SPR would not occur, and the observed prevalence (475 patients). The number of the emigrated patients is equivalent to 15% (83/558) of the patients that would have otherwise resided in distant areas.

**Figure 3:**
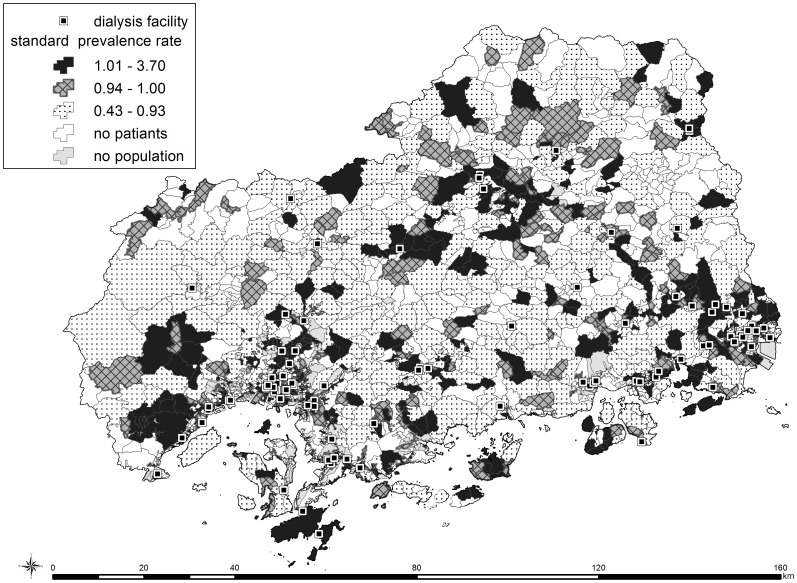
Empirical Bayesian estimate of age- and sex-adjusted standard prevalence rates (SPRs) of dialysis patients at each census block.

Figure 3 shows the empirical Bayesian estimate of age- and sex-adjusted SPR of dialysis patients at each census block. High SPRs are observed at areas surrounding the areas in which dialysis facilities are located. It is noted that some high SPR areas were also found in islands.

The sensitivity analysis revealed that even in a model in which commuting facilities were identified differently, almost identical results were obtained as in the original capacity-distance model, although the effects of travel time were slightly attenuated.

## Discussion

The results of this study revealed that the prevalence rate of dialysis patients substantially decreased with the increase in travel time between the residential area and the commuting dialysis facility; this suggests that travel time serves as a geographic barrier to commuting for dialysis patients. This finding was consistent with previous studies in Wales [7] and in England [4], [6], which suggests that this phenomenon might be observed internationally. The effect of travel time on prevalence of dialysis patients was obvious only on the mainland, but not on the islands.

Reasons for the decreased prevalence among remote residents are still unclear. Authors of previous studies in the UK speculated that it was caused by higher mortality and/or higher rate of withdrawal from dialysis therapy in remote areas [6], [7]. Although mortality might be a reason [26]–[28], it is plausible that patients in distant areas emigrate to more convenient areas in order to shorten the commuting time to the facility. In Japan, a previous study reported that some dialysis patients (43%) need support from their family for transportation to facilities [29]. Furthermore, over half of dialysis patients (52.2%) had undergone dialysis for more than 5 years [10]. During such a long period in therapy, long commuting times can be a burden for patients and their families–much more than in European and North American countries [30]. Cost of transportation for patients living in distant areas might be an access barrier for the patients, although the renal disabled can receive a financial aid from the government for their commuting costs. These senses of burdens might encourage distant patients to move closer to dialysis facilities. We estimated that as many as 15% of distant patients emigrated from their original areas seeking greater convenience for their therapy. Whatever reason is correct, the decreased prevalence of dialysis in distant areas requires political attention. Both high mortality and emigration indicate a serious inequity in health and healthcare within the prefecture. Maintaining facilities in rural and remote areas would be important for preventing a further widening of the accessibility gap among patients [17].

Remote areas have a problem with increasingly feasible public transportation infrastructure or support system for their transportation to dialysis facilities. The number of daily bus departures is lower in areas remote to dialysis facility than that in areas closer (average number of bus departures in the census area: 16.7 vs. 9.2). Although we could not obtain the actual usage rate of those transportations for dialysis patients in this study, most of the dialysis patients generally use cars or buses to commute to facilities in Japan. Age of dialysis patients would be older (i.e., 58% of all the dialysis patients in Japan were 65 years old or older, and 43% were 70 years or older) [10]. Older people generally have difficulty driving a car by themselves. Public transportation or support system for their commuting to dialysis facilities such as pickup buses for the patients would be an important resource for older people, especially in the areas that have less transportation infrastructure.

On islands, the SPR did not change between distant and close areas. On islands, ship was the only way to commute to facilities for 78 of 181 dialysis patients, and it took 46 min on average (minimum: 24 min; maximum: 54 min). The effects of total travel-time on the prevalence might be different from the mainland.

There are some limitations in the present study. First, present subjects potentially included people that had reduced kidney function, but had not yet started dialysis therapy. Although the exact number of dialysis patients among the certified renal disabled is unknown, creatinine clearance level of the study subjects had to be less than 20 mL/min. Those subjects would require renal replacement therapy in a short time even if they did not currently require therapy. The dialysis patients in Hiroshima prefecture in 2011 were reported as 7,202 by the Japanese Society for Dialysis Therapy [10], which is close to this study’s subjects: 7,374. If we assume Japanese Society for Dialysis Therapy captured all the patients, (7,374–7,202)/7,374 = 2.3% of our subjects are not on dialysis. We should note that the number of patients in the Society’s report did not contain a small proportion of patients at some of the facilities (capture rate of facilities was 98.8%) [10]. Thus, the actual number of dialysis patients in Hiroshima prefecture would be closer to the number of our subjects: 7,374. In addition, present subjects might include some people that had received kidney transplantations. However, the rate of kidney transplantations among end-stage renal diseases is only 3% in Japan [11]. Thus, the proportion of transplant patients among the study subjects would not have substantially affected the results.

Another limitation is that the study subjects contained patients undergoing both haemodialysis and peritoneal dialysis. The proportion of patients undergoing peritoneal dialysis among all dialysis patients in Hiroshima was reported at 5.8% [10]. We should note that the burden of commuting to a dialysis facility for patients with peritoneal dialysis would be less than those undergoing haemodialysis. However, in addition to the small proportion of these patients among the subjects, some patients with peritoneal dialysis in Japan are on both peritoneal dialysis and haemodialysis (i.e., 15.2% of those are undergoing peritoneal- and haemo-dialysis once per week, and 2.5% of those are undergoin peritoneal- and haemo-dialysis twice per week) [10].

Finally, and most importantly, misclassification of residential location might have taken place. From our experience, there are a substantial number of patients who, in reality, emigrated to areas closer to dialysis facilities but have not changed municipalities in which they are registered as residents. These patients were treated in the current study as those that live in distant areas. Furthermore some of the patients might be admitted to a hospital. Due to these misclassifications, the effects of travel time on dialysis prevalence seen in this study were probably underestimated. Furthermore, presently estimated travel time was not identical to actual travel time. Although we obtained similar trends in sensitivity analysis using a different capacity-distance model, the real influence of those potential limitations were unclear. A direct patient-interview method is required in further studies to evaluate precisely the emigration of patients.

In conclusion, the present study provides evidence that the prevalence of dialysis patients is lower in areas whose travel time to dialysis facilities are longer. Geographic difficulties for commuting seem to decrease patients’ utilization of dialysis services.

### Ethics Statement

The data in this study was collected by local governments and used in anonymous form with permissions from the governments. This study was approved as a study that can be conducted without individual informed consent by the Ethics Committee of Epidemiological Research, Hiroshima University.

## Supporting Information

Figure S1 **Predicted age- and sex-adjusted standard prevalence rates by land type in capacity-distance model.**
(PDF)
