# Supplementary material for: The Impact of Travel Time on Geographic Distribution of Dialysis Patients
Source: PLoS One. 2012 Oct 17;7(10):e47753. doi: 10.1371/journal.pone.0047753 (PMC3474791; doi:10.1371/journal.pone.0047753)
Supplement: Figure S1 — Predicted age- and sex-adjusted standard prevalence rates by land type in capacity-distance model. (PDF) [file pone.0047753.s001.pdf]

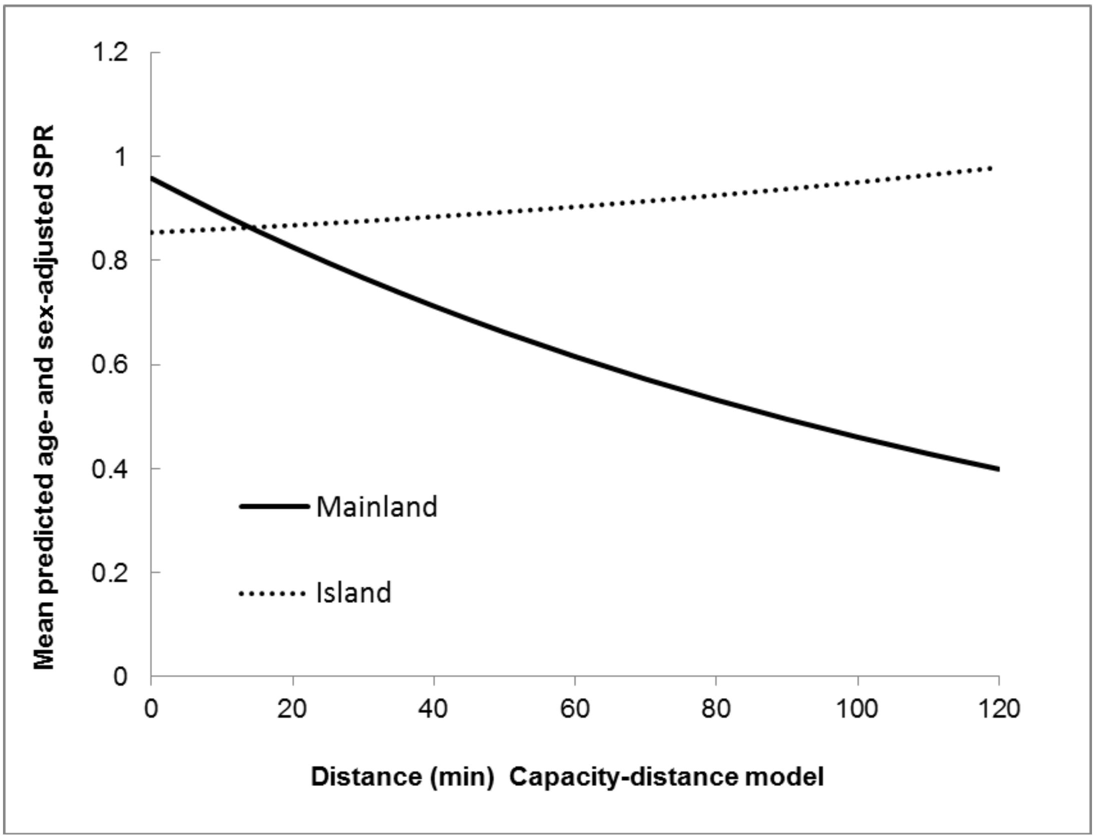

Figure S1. Predicted age- and sex-adjusted standard prevalence rates by land type in capacity-distance model
